# Trial by trial dependencies in multisensory perception and their correlates in dynamic brain activity

**DOI:** 10.1038/s41598-018-22137-8

**Published:** 2018-02-27

**Authors:** Stephanie J. Kayser, Christoph Kayser

**Affiliations:** 1 Department for Cognitive Neuroscience, Faculty of Biology, Bielefeld University, Universitätsstr. 25, 33615 Bielefeld, Germany; 2 Cognitive Interaction Technology – Center of Excellence, Bielefeld University, Inspiration 1, 33615 Bielefeld, Germany; 3 Institute of Neuroscience and Psychology, University of Glasgow, Glasgow, UK

## Abstract

A well-known effect in multisensory perception is that congruent information received by different senses usually leads to faster and more accurate responses. Less well understood are trial-by-trial interactions, whereby the multisensory composition of stimuli experienced during previous trials shapes performance during a subsequent trial. We here exploit the analogy of multisensory paradigms with classical flanker tasks to investigate the neural correlates underlying trial-by-trial interactions of multisensory congruency. Studying an audio-visual motion task, we demonstrate that congruency benefits for accuracy and reaction times are reduced following an audio-visual incongruent compared to a congruent preceding trial. Using single trial analysis of motion-sensitive EEG components we then localize current-trial and serial interaction effects within distinct brain regions: while the multisensory congruency experienced during the current trial influences the encoding of task-relevant information in sensory-specific brain regions, the serial interaction arises from task-relevant processes within the inferior frontal lobe. These results highlight parallels between multisensory paradigms and classical flanker tasks and demonstrate a role of amodal association cortices in shaping perception based on the history of multisensory congruency.

## Introduction

We usually perform better in perceptual tasks when the relevant information appears in multiple sensory modalities. In particular, congruent information presented to two senses usually leads to faster and more accurate responses when compared to incongruent multisensory evidence^1^. While such multisensory congruency effects are well known recent studies have shown that perception is also affected by the multisensory composition of stimuli experienced during the preceding trials. For example, the perceived simultaneity or location of audio-visual stimuli depends not only on the currently presented stimuli but also on the asynchrony or spatial position experienced on previous trials^2–9^. While some studies have started to investigate the computational underpinnings of such trial-by-trial dependencies in multisensory perception (e.g.^3,6,10,11^) we still know little about the relevant neural mechanisms and brain networks.

Understanding the origin of trial-by-trial dependencies in multisensory perception becomes even more important in the light of similar history dependent effects in unisensory perception^12^. Trial-by-trial dependencies have been well documented for near threshold or ambiguous visual stimuli^13,14^, and more generally, are well known in unisensory interference paradigms, such as flanker or Stroop tasks^15^. In these tasks the congruency of task-relevant and distractor stimuli interacts across trials (e.g. the Gratton effect)^16–19^: congruency effects (arising from the stimuli presented on the current trial) on response accuracy and reaction times are usually more pronounced following a previous congruent than following a previous incongruent trial. The ubiquity of trial-by-trial interactions in unisensory and multisensory paradigms raises the question as to whether and which of these trial-by-trial dependencies arise from mechanisms that are unique to multisensory perception, and which rather reflect generic and amodal processes.

There are many parallels between classical interference tasks and multisensory paradigms, which have not been explored. While multisensory studies often ask subjects to pay attention to both sensory modalities it remains uncertain whether the attentional load is indeed fairly divided between the senses^20,21^. As a result, many studies are designed around a single task-relevant modality (so called focused attention), and rely on the automatic association of sensory information across the modalities to characterize multisensory processes (e.g.^22–24^). For example, the detection or discrimination of visual motion direction is enhanced by auxiliary acoustic information, even when subjects are instructed to focus on a visual task^25–27^. This multisensory paradigm is generally equivalent to unisensory interference tasks, where one sensory item is task-relevant and other items are not: e.g. the direction of a central arrow can be task relevant, while the direction of surrounding arrows nevertheless shapes performance depending on the congruency with the central item^28^.

We here capitalize on these parallels between multisensory and flanker paradigms to ask whether the well-known serial dependency known from interference tasks is also observed in a multisensory paradigm. In particular, our main aim was to understand whether the putative neural correlates of such a serial effect in multisensory decision making are the same or different from the better-known neural correlates of multisensory congruency, which are observed when contrasting trials with congruent and incongruent multisensory information. To this end we re-analysed behavioural and EEG data from an audio-visual motion discrimination task for which we have previously localized the dynamic neural correlates of multisensory congruency to sensory-specific visual cortices^27^. We first verified that a serial interaction of multisensory congruency indeed emerges for response accuracy and reaction times. We then used single-trial analysis of EEG data to localize and compare the neural correlates of current-trial congruency and serial interactions.

## Materials and Methods

The data analysed here have been published previously^27^. They were obtained from 18 healthy adult participants (8 males; mean age of 21.3 years) with self-reported normal hearing and vision. The study was conducted in accordance with the Declaration of Helsinki, was approved by the local ethics committee (College of Science and Engineering, University of Glasgow), and each subject provided informed consent before participating.

### Experimental design and procedure

**Figure 1:**
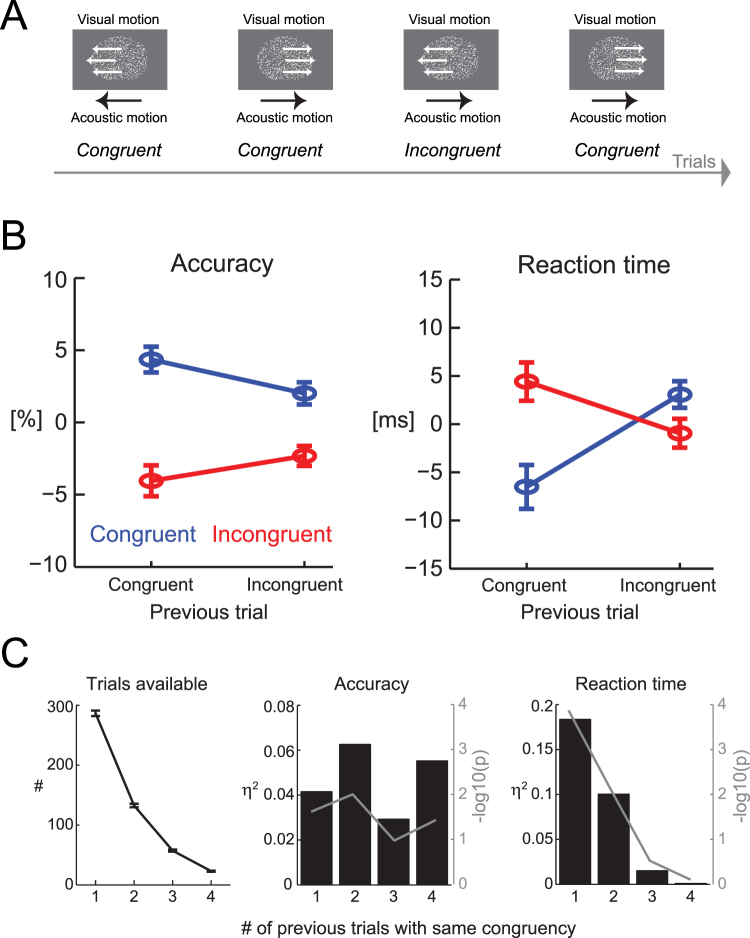
Behavioural data. (**A**) Subjects discriminated the direction of visual motion in random dot displays that were accompanied by acoustic motion moving either in the same (congruent) or opposite (incongruent) direction. The directions of motion and the multisensory congruency varied pseudo-randomly across trials. (**B**) Effects of current-trial (color-coded) and previous-trial (x-axis) congruency on accuracy and reaction times. For visualization the data were normalized to zero-mean within each subject to remove between subject-variability. (**C**) Serial-interactions for trials with one or more preceding trials of the same congruency. Bars display the statistical effect size (η^2^), lines the log-transformed p-values.

Subjects performed a discrimination task on random dot displays containing left or right-wards visual motion (Fig. 1A). Motion coherence varied across four levels and stimuli lasted 1.2 s (preceded by 0.7–1.1 s uniform fixation periods; 1.5–2 s uniform inter-trial intervals). For each subject the four coherence levels were defined as [0.55, 0.85, 1.15, 1.45] x the subject-specific coherence threshold, which had been defined in a separate session at a criterion of 71% correct. Random dot displays covered 15° of visual angle and were presented on a 21′ CRT monitor at a refresh rate of 85 Hz. Visuals stimuli were accompanied by an acoustic stimulus mimicking motion in either the same (audio-visual congruent) or the opposite (audio-visual incongruent) direction as the visual stimulus. Acoustic motion was created by linearly modulating the amplitude of white noise between the two ears over the stimulus period. Sounds were presented with a peak amplitude of 65 dB SPL r.m.s. level via headphones. Stimulus presentation was controlled from Matlab (Mathworks) using routines from the Psychophysics toolbox^29^.

Each subject performed 1200 trials, 150 for the each of the eight experimental conditions (4 coherence levels × 2 levels of audio-visual congruency). Subjects were instructed ‘to discriminate the direction of visual motion and to respond as quickly and accurately as possible and to ensure they responded within the stimulus period’. In an initial training block subjects received feedback on response time (negative feedback when responding after stimulus offset) and feedback on performance. During the actual task no feedback was provided.

EEG signals were recorded using an active 64 channel BioSemi system (BioSemi, B.V., The Netherlands), with additional electrodes placed near the outer canthi and below the eyes to obtain the electro-occulogram (EOG). Electrode offsets were below 25 mV. For storage the data were sampled at a rate of 500 Hz using a low pass filter of 208 Hz. For more details please see^27^.

### Analysis of EEG data

Data analysis was carried out offline with MATLAB (The MathWorks Inc., Natick, MA), using the FieldTrip toolbox^30^ and custom written routines. The pre-processing of the data was similar as reported in the previous study, and included filtering between 0.2 and 70 Hz, the removal of trials based on amplitude thresholds and de-noising using ICA^27^. Trials with reaction times shorter than 0.3 s or longer than 1.2 s were excluded. For subsequent analyses the EEG signals were referenced to the common average reference, sampled at 150 Hz, and were re-aligned to the reaction time of each trial.

**Figure 2:**
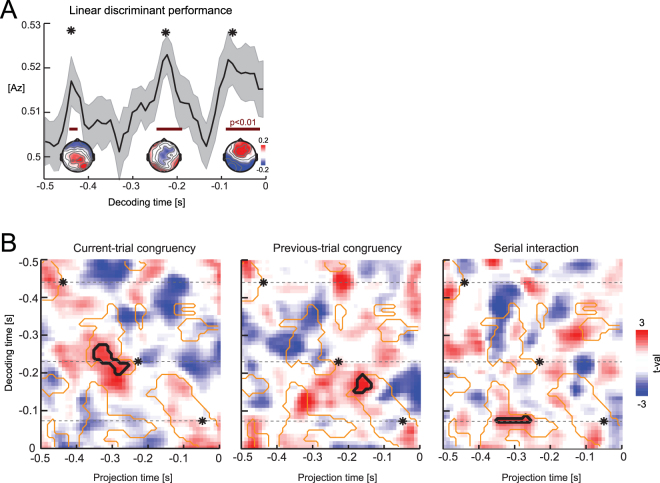
Discriminant analysis of EEG data, aligned to response time. (**A**) Performance of the linear discriminant characterized by Az (mean and s.e.m. across subjects). Epochs with significant performance are indicated in red (cluster-based permutation statistics, p < 0.01 FWE along time). Scalp topographies are shown for three peaks (*). (**B**) Statistical tests for each congruency effect in the two-dimensional time-domain defined by the time at which each EEG discriminant was defined (‘decoding time’) and the time at which the projection of this component was evaluated (‘projection time’). Images show color-coded group-level t-values. Black outlines denote significant congruency effects (cluster-based permutation statistics, p < 0.05 FWE), orange outlines projections with a significant predictive value for single trial choice (p < 0.05, FWE). Note that we tested for congruency effects only within significant choice-predictive epochs. * denote the time points of the three peaks in the discriminant performance (c.f. panel A), while dashed lines denote their projections along the entire trial. These three discriminant projections are defined based on EEG components characterized by significant task-relevant visual-motion information.

For single trial analysis we used a regularized linear discriminant to extract EEG components sensitive to the task-relevant stimulus feature, the direction of visual motion^31^. For this analysis the data were filtered between 1 and 25 Hz (3^rd^ order Butterworth filter). For a given time t during the trial the discriminant was calculated across all levels of motion coherence and both audio-visual congruencies, and is defined by a projection vector (w) describing a one dimensional combination (y) of the EEG data x(t):

1 $${\rm{y}}({\rm{t}})=\sum _{{\rm{i}}}{{\rm{w}}}_{{\rm{i}}}{{\rm{x}}}_{{\rm{i}}}({\rm{t}})+{\rm{c}}$$

with i summing over all electrodes, c being a constant, and the EEG data being averaged over 60 ms time windows. Classification performance was quantified using the receiver operator characteristic (Az) based on 6-fold cross validation. We used trial sub-sampling to ensure that equal trial numbers per category were used to establish the discriminant (using 80% of the minimally available trials per condition, repeating the analysis 100 times). Single trial projections of each discriminant component defined at a specific time point (termed ‘decoding time’ in the following) were obtained by applying the weights, w, to all trials and all time points within each trial. This resulted in a two-dimensional analysis space, defined by the decoding time at which the discriminant was established, and a ‘projection time’ at which the single trial projection was evaluated (Fig. 2). As shown previously, linear discriminant components can provide a proxy to the neural representation of the underlying task-relevant information^27,31^. Importantly, when defined based on discriminant components carrying significant task-relevant information (as defined by a significant ROC value) they can exhibit a typical ramping behaviour, whereby the amount of motion evidence reflected by the discriminant rises slowly in the period before the decoding time point^27,32^. We furthermore tested the relevance of each discriminant component for predicting subjects’ performance by entering the discriminant value (Eq. 1) together with the actual direction of visual motion into a logistic regression of choice. A significant contribution of the discriminant to choice indicates a significant trial-by-trial co-variation of the visual motion information contained in the EEG signal and the subject’s response.

**Figure 3:**
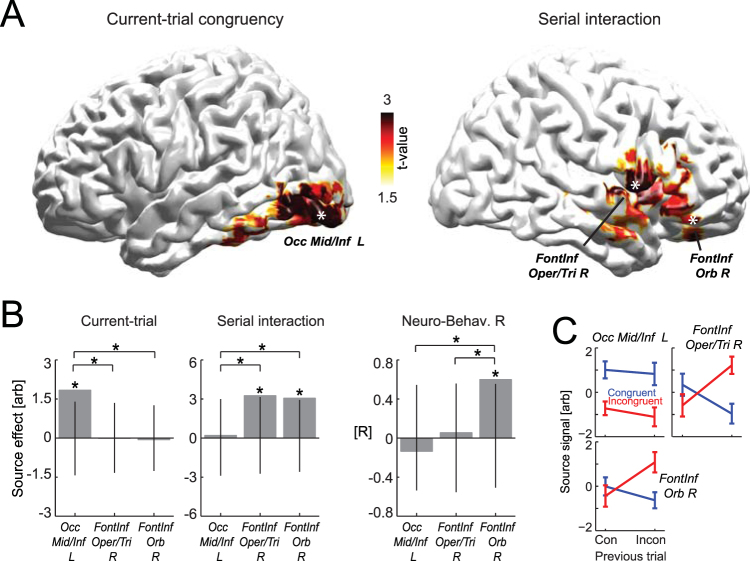
Analysis in source space. (**A**) Statistical test for current-trial and serial effects (at t = −0.3 s prior to the response) in source space, with significant clusters color-coded (at p < 0.05, FWE across voxels). (**B**) Effect size (group-level mean) of each congruency effect and neuro-behavioural correlations of the serial effect at three peak ROIs (one from the occipital cluster; two from the inferior frontal cluster; indicated by white * in panel A; see Table 1 for anatomical locations). We performed post-hoc percentile bootstrap tests for the significance of each effect against zero (black lines indicate the 99^th^ percentile confidence intervals) and the effect difference between ROIs (indicated by connecting lines) to directly support the conclusion that distinct congruency effects originate from distinct brain regions. * denote significant effects (at least p <= 0.05). (**C**) Source activity within the three ROIs (mean and s.e.m. across subjects).

**Figure 4:**
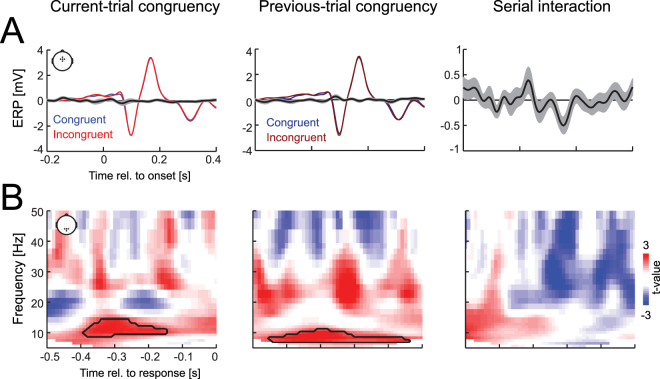
Congruency effects in ERPs and oscillatory activity. (**A**) Congruency effects in fronto-central ERPs when aligned to stimulus onset. Coloured lines indicate group-level means for individual conditions, black lines (grey outlines) the mean (s.e.m.) for the respective ERP differences. When tested across all electrodes and time points there were no significant effects (cluster-based permutation statistics; FWE p < 0.05). (**B**) Congruency effects in parietal oscillatory activity, shown by color-coded group-level t-values. Effects of current-trial and previous-trial congruency revealed significant clusters in the alpha band (black outlines; cluster-based permutation statistics; FWE p < 0.05); there was no significant serial interaction.

Single trial source signals for the analysis in Fig. 3 were derived using a linear constrained minimum variance beamformer (LCMV, 7% normalization, using the covariance matrix obtained from −0.7 to −0.1 s prior to response, projecting along the dominant dipole orientation) as implemented in the FieldTrip toolbox. As subject-specific anatomical data were not available, we relied on a standardized head model using the average template brain of the Montreal Neurological Institute. Lead-fields were computed using a 3D grid with 6 mm spacing. Evoked responses for the analysis in Fig. 4 were calculated based on the data filtered between 0.5 and 30 Hz (3^rd^ order Butterworth filter). Time frequency (TF) representations of the EEG sensory data in Fig. 4 were obtained between 4 and 80 Hz, in steps of 1 Hz below 16 Hz and steps of 2 Hz above, using a 5 Hz wavelet width. Trial-averaged TF representations were baseline normalized to a pre-trial period (−0.5 to −0.1 s before stimulus onset). We here only focused on occipito-parietal electrodes of interest (PO3, PO4, Pz, POz), as we had previously described congruency effects for these^27^.

### Statistical analyses

In the previous study^27^ we had investigated effects of audio-visual congruency (based on the current-trial) in the behavioural data and for two specific discriminant components extracted from the EEG data. In the present study we systematically investigated the influence of both current-trial and previous-trial congruency, as well as their interaction, systematically on behavioural and EEG data. The analysis of behavioural data was based on a 2 × 2 repeated measures ANOVA, averaging performance across coherence levels. EEG activity was investigated in the discriminant space, defined by each discriminant component (established at a specific decoding time) and its projection during the trial (Fig. 2). Cluster-based randomization statistics correcting for multiple comparisons along time was used to test for each congruency effect in the EEG data (detailed parameters: 2000 iterations; clustering bins with individual p < 0.05; minimal cluster size of at least 8 neighbours; computing the summed cluster-mass; performing a two-sided test)^33,34^. Clustering was applied to group-level t-values of the respective contrast of interest. This was either i) the difference between congruent – incongruent trials, ii) the difference between trials for which the previous trial was congruent or incongruent, or iii) the interaction effect defined as the congruency difference (congruent – incongruent) for trials on which the previous trial was congruent, minus the congruency difference for trials on which the previous trial was incongruent. In each case, the effects were averaged across coherence levels for each individual subject. The same cluster-based approach was used for testing the significance of choice prediction of each discriminant component; here group-level t-values were derived from single subject regression betas. Given the concern of sample sizes lower than 20^35^ and the use of possibly too lenient cluster forming thresholds in neuroimaging analysis^36,37^, we performed additional simulations to verify that the implemented statistical tests for congruency effects did not result in an inflated false positive rate. For a set of 1000 randomized group samples we calculated the familywise error rate in detecting significant congruency effects (of either type) based on the precise parameters and thresholds used for the actual analysis^36^. This revealed that the FWE for detecting false positive congruency effects within the mask defined by significant choice productiveness was 1.3%.

The analysis of congruency effects in source space (Fig. 3) was similarly based on a cluster-based permutation procedure (1000 iterations; clustering bins with individual p < 0.05; minimal cluster size of at least 8 neighbours; computing the summed cluster-mass; performing a two-sided test clustered data). The correlation of the interaction effect between source activity and behavioural accuracy was defined as their Pearson correlation. Finally, we calculated confirmatory statistics for the peak ROIs derived from the source analysis using the percentile bootstrap. Here we derived confidence intervals for congruency effects and the neuro-behavioural correlation within each ROI tested against the null-hypothesis of a zero effect. In addition, and to support the distinct localization of current-trial and serial effects to distinct brain regions, we tested the difference effect between ROIs against zero using the percentile bootstrap to derive confidence intervals for the difference. For the comparison of effects across ROIS in Fig. 3 p-values were corrected using the Benjamini & Yekutieli procedure^38^.

### Data availability

The datasets generated during and/or analysed during the current study are available from the authors on reasonable request.

## Results

### Behavioural data

As reported previously^27^ subjects’ performance in discriminating the direction of visual motion was higher during congruent compared to incongruent audio-visual trials (Fig. 1). As our main interest was the influence of the multisensory congruency experienced in the previous trial on performance in the subsequent trial we analysed the data using a 2 × 2 ANOVA. For accuracy this revealed a significant effect of current-trial congruency (n = 18 subjects; F(1,71) = 54.0, p < 10^−3^, η^2^ = 0.42; Fig. 1B), no effect of previous-trial congruency (F = 0.12, p = 0.72, η^2^ = 0.001), and a significant interaction (F = 5.5, p = 0.02, η^2^ = 0.05). Reaction times varied between 0.44 and 0.82 s (trial-median) across subjects, with an overall group-level median of 0.66 s. For reaction times there was no effect of current-trial congruency (F(1,71) = 3.5, p = 0.06, η^2^ = 0.04), no effect of previous-trial congruency (F = 1.3, p = 0.25, η^2^ = 0.01), but a significant interaction (F = 16.7, p < 10^−3^, η^2^ = 0.19).

This serial (i.e. trial-by-trial) interaction of multisensory congruency persisted when tested over longer periods of same-type-congruency exposure on previous trials (Fig. 1C). Prolonged exposure (up to 4 trials) of all congruent (or incongruent) previous trials consistently induced a significant statistical interaction in response accuracy regardless of the number of preceding trials (c.f. p-values and effect sizes in Fig. 1C). For reaction times the effect disappeared with more preceding trials of the same type congruency and the interaction was no longer significant for 3 or 4 preceding trials of a given congruency type.

We also analysed behavioural responses as a function of the accuracy on the previous trial. Post-error slowing is a well-known effect and it could be that previous accuracy affects the degree to which multisensory congruency influences performance. Such an effect is not implausible, given that performance was generally higher and somewhat faster on congruent trials. Separating trials based on accuracy on the previous trial and on congruency on the current trial revealed a significant effect of congruency (F(1,71) = 45.0, p < 10^−3^, η^2^ = 0.38), no effect of previous performance (F = 3.1, p = 0.08, η^2^ = 0.026), and no interaction (F = 1.1, p = 0.29, η^2^ = 0.009). For reaction times this revealed no effect of congruency (F = 0.68, p = 0.42, η^2^ = 0.006), a significant effect of previous performance resulting from faster responses following a correct trial as expected based on post-error slowing (F = 47.2, p < 10^−3^, η^2^ = 0.40), and no interaction (F = 0.11, p = 0.73, η^2^ = 0.001). This suggests that the serial interaction of congruency is not a direct consequence of performance changes associated with multisensory congruency on the previous trial.

### Multisensory congruency effects on task-relevant EEG components

Using linear discriminant analysis, we mapped the EEG components carrying task-relevant sensory information, i.e. we searched for components that discriminated significantly between the two directions of visual motion (Fig. 2). To account for variations in reaction times between participants we applied the discriminant analysis to EEG activity aligned to the response. Consistent with our previous study we found multiple components with significant discriminant performance (n = 18 subjects; p < 0.01 FWE corrected along time, two-sided cluster-based permutation test; Fig. 2A). These peaked around t = −0.45 s, t = −0.23 s, and t = −0.08 s prior to the response, and were each characterized by a distinct scalp projection (Fig. 2A insets).

To understand when and where the EEG activity reflects effects of multisensory congruency, we used the discriminant weights obtained at each time point to define a projection of the respective EEG activity along the trial. These projections characterize the temporal profile of task-relevant sensory representations of visual motion direction as detectable via the EEG signal. We then analysed the single-trial projections of the discriminant time course to test for statistical congruency effects. The resulting two-dimensional contrast maps in Fig. 2 display each congruency effect as a function of the time prior to the response at which the linear discriminant weights were defined (‘decoding time’, i.e. the time at which the linear discriminant was established; Fig. 2B), and the time at which the respective discriminant projection was tested for congruency effects (‘projection time’). Given that we were only interested in effects that are directly relevant for shaping behaviour, we searched for significant congruency effects within a mask defined by a significant choice prediction of the respective EEG component (at p < 0.05 FWE corrected, two-sided cluster-based permutation test; Fig. 2B; orange outlines). Consistent with our previous study, we found significant effects of current-trial congruency within the discriminant component defined around t = −0.23 s, with a cluster of a significant congruency effect between t = −0.35 and t = −0.26 s prior to the response (Fig. 2B black outline; T_sum_ = 120, p < 10^−3^, two-sided cluster-based permutation test, n = 18). The positive sign of the effect indicates that the amount of evidence about visual motion direction contained in the EEG activity was higher during congruent compared to incongruent trials. Given that across subjects median RTs were around 0.66 s, this congruency effect arises at about 0.3 s post-stimulus onset.

We also found a significant effect of previous-trial congruency, which emerged between −0.18 s and −0.14 s prior to the response (T_sum_ = 50, p = 0.01; defined at decoding time of t = −0.17 s). However, this effect arose from a projection of discriminant component which itself did not carry significant visual motion evidence (c.f. Fig. 2A; lack of significant Az at t = −0.17 s). As a result, this effect remains difficult to interpret in functional terms, as the underlying EEG component is not characterized by task-relevant information.

Finally, we found a significant serial interaction of multisensory congruency. This emerged within the projection of the discriminant component defined just prior to the response (decoding time t = −0.08 s; projection time t = −0.35 s to t = −0.26 s; T_sum_ = 27, p < 10^−3^). Hence the serial interaction emerged within an EEG component characterized by significant task-relevant information. Noteworthy, the interaction effect localized around the same time during the trial as the current-trial congruency effect.

Our approach of testing for congruency effects within the full range of discriminant components and their projections throughout the trial did not make a priori assumptions of whether current-trial and serial interactions should emerge at the same time during the trial. Our findings hence genuinely demonstrate that effects of current-trial congruency and serial interactions emerge from distinct task-relevant EEG components, hence likely reflect distinct neural generators, but co-exist around the same time during the trial.

### Localizing EEG activations in source space

To better understand the brain regions from which the different congruency effects arise we performed a source analysis. More specifically, having constrained the emergence of current-trial and serial effects to the same epoch during the trial, we systematically tested source activity at this time (t = −0.3 s). This revealed significant clusters of current-trial congruency effects in occipital cortex (Fig. 3A; Table 1; p < 0.05 FWE corrected, two-sided cluster-based permutation test), consistent with the previous and technically slightly different analysis of this data. Importantly, a cluster with a significant serial interaction emerged in the right frontal lobe, spanning from the inferior frontal pars opercularis and pars triangularis to the pars orbitalis (Table 1).

**Table 1:** Congruency effects in source space.

**Effect**	**Cluster T**	**P**	**Atlas Label**	**MNI**
Current-trial	540	p = 0.017	Occ Mid/Inf L	[−35 −86 −11]
Serial interaction	742	p = 0.019	Front Inf Oper/Tri R;	[49 9 5]
			Front Inf Orb R;	[56 27 −8]

The table lists local and global peak values within the statistically significant clusters in source space, including effect size, peak locations and anatomical labels from the AAL atlas^65^.

To further corroborate that effects of current-trial congruency and serial interactions emerge in distinct parts of the brain we performed a post-hoc bootstrap analysis on the respective peak sources. One ROI was selected as the global peak effect within the occipital cluster, and two ROIs were defined based on local peaks within the frontal cluster (c.f. Table 1). The post-hoc analysis revealed a significant influence of current-trial congruency only at the occipital (Occ Mid/Inf L p = 0.009 FDR corrected across ROIs and effects, n = 18, two-sided percentile bootstrap; c.f. Fig. 3B) but not the two frontal ROIs when each was contrasted against a null effect size (Front Inf Oper/Tri p = 0.602, Front Inf Orb p = 0.702). Effects of serial interactions emerged only at the two frontal (Front Inf Oper/Tri p = 0.02, Front Inf Orb p = 0.021) but not the occipital ROI (Occ Mid/Inf L p = 0.614). Furthermore, and to directly ascertain that current-trial and serial effects dominate in distinct regions, we contrasted effect sizes between ROIs (Fig. 3B). This revealed that the current-trial effect was indeed significantly stronger in the occipital ROI (against Front Inf Oper/Tri p = 0.017, against Front Inf Orb p = 0.014), while the serial interaction was significantly stronger in the two frontal ROIs compared to the occipital ROI (Front Inf Oper/Tri p = 0.045, Front Inf Orb p = 0.05).

Finally, we asked whether the effect of serial-congruency seen in the source activity correlated with the corresponding behavioural effect across subjects. This neuro-behavioural correlation was significant only for the inferior frontal orbital ROI (Front Inf Orb p = 0.013; Front Inf Oper/Tri p = 0.602; Occ Mid/Inf p = 0.704, two-sided Pearson correlation), and the difference between this and the other frontal (p = 0.004) and the occipital ROI was significant (p = 0.007).

### No ERP signatures of conflict

For comparison with established EEG correlates of response conflict^39–41^, we investigated the evoked responses computed relative to stimulus onset (Fig. 4). Typical conflict potentials arise between 200 and 600 ms post-stimulus onset. However, we here could only analyse the time window up to 400 ms, given that the shortest median reaction times of individual subjects were around 450 ms. We used a hypothesis free cluster-based permutation procedure to detect statistically significant effects of each congruency. This revealed no significant effects (p < 0.05 FWE corrected, two-sided cluster-based permutation procedure). Figure 4A illustrates the evoked responses for fronto-central electrodes.

### Alpha band correlates of multisensory congruency

For comparison with previous studies implying a role of alpha band activity and possibly related attentional processes in multisensory perception^27,42^, we tested for congruency effects in oscillatory activity. Motivated by these previous studies we focused on parieto-occipital electrodes of interest (Fig. 4B). This revealed a significant effect of current-trial congruency within the alpha band (T_sum_ = 250, p = 0.026, between −0.39 s and −0.15 s, centred around 11 Hz, two-sided cluster-based permutation procedure, FWE corrected), as well as a significant effect of previous-trial congruency (T_sum_ = 317, p = 0.006, between −0.45 s and −0.08 s, centred around 9 Hz), but no significant serial interaction.

## Discussion

Our results show that the influence of multisensory congruency on perceptual decisions is shaped by the nature of the multisensory congruency experienced on previous trials. While subjects generally responded faster and more accurately when exposed to congruent audio-visual stimuli, this congruency benefit was reduced when subjects had previously experienced an incongruent rather than a congruent audio-visual stimulus. Importantly, by providing a comparative analysis of current-trial and serial interaction effects on task-relevant EEG components we reveal that these arise from distinct neural origins. While the multisensory congruency experienced during the current trial influences the encoding of task-relevant information in sensory-specific visual brain regions, the serial effect originates from the inferior frontal lobe. These results pave the way to better understand the link between trial-by-trial dependencies in multisensory perception and the general literature on sensory-response conflict during perceptual decision making.

### Serial interactions in multisensory decision making

It is well known that perception is influenced by the congruency of multisensory information, with subjects typically responding faster and more accurately when exposed to congruent information across the senses. In addition, several studies have shown that perception is also influenced by the multisensory properties of stimuli experienced on previous trials. For example, the point of perceived simultaneity adapts to the previously experienced multisensory asynchrony^2–5,43^. Similarly, the localization of audio-visual stimuli is influenced not only by each sense’s reliability and the potential disparity between acoustic and visual information, but also by the experienced and perceived locations on the previous trial^6–9^. Our results extend this literature by demonstrating that serial interactions also exist for judgements of motion direction.

Several studies have investigated the neural and computational mechanisms underlying multisensory trial-by-trial dependencies^11,44^. Importantly, many studies did not quantify the congruency effect itself, as studied here, but focused on shifts in the reported feature values, such as the perceived timing or spatial position. While one study suggested that multisensory recalibration results from changes in sensory- specific representations rather than more basic mechanisms such as desensitization^10^, a more principled model-based approach failed to find a coherent explanation for temporal recalibration effects^11^. In contrast, a Bayesian study on spatial recalibration reported that changes in the perceived location are best explained by a shift in the probabilistic representation of spatial evidence rather than a change in precision of this representation or a change in a priori bias^6^. This would suggest a mechanistic origin in sensory-specific cortices rather than amodal regions implementing behavioural choice. This interpretation again is in contrast to an EEG study reporting long-latency correlates of recalibration effects in evoked potentials^44^. One potential explanation for these discrepancies is that multisensory recalibration may emerge independently at multiple time scales, suggesting that multisensory decisions can be prone to multiple and possibly functionally distinct history-dependent effects^45^.

The use of a two-response paradigm in the present study did not allow us to investigate fine quantitative changes in the sensory representation of visual motion direction. As a result, we cannot differentiate specific computational underpinnings of the serial interaction, such as a change in the precision of task-relevant neural representations from a decision-related effect. It remains to be studied whether the same or distinct computational mechanism are responsible for the effects observed here and in previous studies on recalibration^3,5–7^. At the same time, however, our data draw parallels to classical unisensory interference tasks by revealing that well-known trial-by-trial effects of congruency (i.e. the Gratton effect) emerge also in multisensory paradigms. Hence, the present data highlight important parallels between perceptual decisions in multisensory paradigms and generic congruency effects as known from interference tasks^16–19^. Given that congruency effects arising from the current trial did not interact with the overall performance on the previous trial our results suggest that the described serial interaction has an origin different from post-error slowing^46,47^.

### Distinct origins of current-trial and serial congruency effects

Multiple accounts for the serial effect in unisensory flanker tasks have been proposed, including increased attention following incongruent trials^16^ or changes in stimulus-response priming that are modulated by the previously experienced congruency^18,48^. While the present study was not designed to disentangle potential mechanisms underlying this interaction, the distinct neural sources of current-trial and serial congruency provide several important insights.

Confirming our previous report, we found that the effect of current-trial congruency is best explained by changes in the neural representation of visual motion direction in occipital cortex. This corroborates previous studies suggesting that multisensory information can enhance the representation of motion direction in hMT/V5^49–52^. The long latency of the current-trial congruency effect of about 300 ms suggests that this results from top-down feedback rather than a feed-forward convergence of sensory information^53^.

In contrast to this we found that the serial interaction is best explained by activations in the inferior frontal lobe, in particular the orbital part. This localization is supported by the source analysis and the frontal topography of the respective discriminant components. Importantly, the discriminant component giving rise to the serial interaction was distinct from that underlying the current-trial congruency effect in occipital cortex, although both effects emerged around the same during the trial. This demonstrates that the serial interaction does not arise from changes in short-latency effects or from sensory-specific representations, based on which one would rather expect a correlate within sensory-specific occipital brain regions. Rather the post-hoc analysis of peak sources clearly speaks in favour of an origin of the serial interaction within supramodal frontal regions. Such an interpretation is consistent with a recent EEG study on temporal recalibration^44^, which reported ERP correlates of recalibration over fronto-parietal sites at around 300 ms post-stimulus onset^32,54^.

Previous studies on serial effects in decision making have implied the anterior cingulate and the DLPFC in conflict monitoring and adapting sensory processes based on trial history^55–57^. Two EEG signatures of conflict are commonly investigated: the Stroop N450 and a slow conflict potential around 600 ms^39–41^. Noteworthy, the N450 has also been implied in a study on temporal recalibration based on long-term adaptation^58^, while it was absent in a study on trial-by-trial recalibration^44^. Our results do not imply a role of either of these two evoked components in the observed serial effect. First, the timing of the serial effect was around 300 ms post-stimulus onset, which is earlier than either of these components. Second, both the N450 and the slow conflict potential typically exhibit main effects of current-trial congruency, which we did not observe in the frontal source^39,41^. And third, the neuro-behavioural correlation of the serial effect was strongest in the orbital part of the inferior frontal cortex, suggesting a source outside the DLPFC. Yet, we have to acknowledge that the use of speeded responses may possibly induce evoked responses with different latencies than typically observed in non-speeded paradigms. At the same time, we observed no significant congruency effects in stimulus-locked ERPs. All in all, our data hence speak against well-known conflict potentials as the main source of the serial effect, and call for a more fine-grained analysis of the respective neural underpinnings.

Previous work has shown that multisensory integration and attentional selection are intertwined, with attention facilitating the binding across modalities by amplifying the representation of co-occurring objects^25,59–61^ or influencing trial-by-trial recalibration^62^. Furthermore, attention has been considered as one potential mechanism contributing to the Gratton effect^16,18^. Parietal alpha band activity has been linked to visuo-spatial attention and can hence serve as a proxy to attention-related brain activity^63,64^. While we found main effects of current- and previous-trial congruency in the alpha band, there was no significant serial interaction. Furthermore, our previous analysis of the present data suggested that trial-by-trial changes in parietal alpha power were not predictive of fluctuations in behavioural performance^27^. This speaks against an interpretation of attention-related processes as being the main cause of the serial interaction.

## Conclusion

We show that the impact of multisensory congruency experienced on any given trial depends on the nature of the congruency experienced on previous trials. This serial dependency of multisensory congruency links the literature on multisensory perception with studies on sensory-response conflict, raising the question to what degree serial interactions in multisensory and classical interference paradigms arise from a shared neural substrate. Importantly, our data suggest that the serial effect of multisensory congruency arises from ventral frontal regions carrying task-relevant sensory information, and hence support a distinct origin from current-trial congruency effects, which rather arise from sensory-specific occipital brain regions. These results support a hierarchical model of multisensory integration: one in which neural representations in modality-specific regions are modulated by the currently perceived sensory congruency, while the actual decision is further shaped by influences regarding task-demands and the recent sensory experience.
